# Smoking, Obesity, and Metabolic Syndrome in Two High Security Settings

**DOI:** 10.1192/j.eurpsy.2025.1515

**Published:** 2025-08-26

**Authors:** G. Goktas, I. Jeandarme

**Affiliations:** 1 Medical Director; 2 FPC Antwerpen, Antwerp, Belgium

## Abstract

**Introduction:**

Two high security Forensic Psychiatric Centers (FPC) were implemented in the last decade in Flanders (FPC Ghent and FPC Antwerp). FPCs provide court[1]ordered treatment for forensic psychiatric patients (called “internees”) that have committed a criminal offense related to their psychiatric disorder and who are at a high risk for recidivating.

**Objectives:**

Treatment is often mandatory and lengthy, and per[1]sonal rights are highly restricted, which has an impact on treatment motivation and overall patient wellbeing (Lutz et al., 2022). While the Risk Need Responsivity (RNR) model - which focuses on risk reduction – was and still is the prominent model for offender rehabili[1]tation, other aspects are currently given more atten[1]tion. This includes the Good Lives Model (GLM) and the recovery movement, that aim at improving the achievement of skills necessary to maintain a good live and give meaning and value in one’s existence (Lutz et al., 2022).

**Methods:**

Data collection and statistical analyses The descriptive analyses of categorical and continuous variables was done using SPSS version 28. Valid per[1]centages were given. Assumption testing was per[1]formed and found that the data were not eligible for parametric examination. The significance level was set at .05.

**Results:**

Weight and BMI At the initial measurement, the mean weight was 89.3 kg (SD ¼ 21.64, range ¼ 46–222 kg). The mean weight at second measurement was 92.6 kg, with a standard deviation of 21.90 and a range of 46.6–185.6. The second weight measurement occurred 812.2 days or 2.2 years (SD ¼ 552.39, range ¼ 7–3084 days) after the initial measurement. The weight difference between the two measurements ranged from a weight loss of 53.8 kg to a weight gain of 51.6 kg and showed a mean weight increase of 2.2 kg (SD ¼ 10.41)1

**Conclusions:**

The overall conclusion of the study is that obesity, MetS, smoking, as well as substance misuse are highly prevalent among our high security population. These are all risk factors associated with somatic morbidity and mortality. Our study showed that obesity and MetS were found across psychiatric diagnoses and the presence of metabolic syndrome was not limited to the use of SGA.

**Disclosure of Interest:**

None Declared

